# Perception and Cognition in the Ageing Brain: A Brief Review of the Short- and Long-Term Links between Perceptual and Cognitive Decline

**DOI:** 10.3389/fnagi.2016.00039

**Published:** 2016-03-01

**Authors:** Katherine L. Roberts, Harriet A. Allen

**Affiliations:** ^1^Department of Psychology, University of WarwickCoventry, UK; ^2^School of Psychology, University of NottinghamNottingham, UK

**Keywords:** perception, cognition, ageing, audition, vision

## Abstract

Ageing is associated with declines in both perception and cognition. We review evidence for an interaction between perceptual and cognitive decline in old age. Impoverished perceptual input can increase the cognitive difficulty of tasks, while changes to cognitive strategies can compensate, to some extent, for impaired perception. While there is strong evidence from cross-sectional studies for a link between sensory acuity and cognitive performance in old age, there is not yet compelling evidence from longitudinal studies to suggest that poor perception causes cognitive decline, nor to demonstrate that correcting sensory impairment can improve cognition in the longer term. Most studies have focused on relatively simple measures of sensory (visual and auditory) acuity, but more complex measures of suprathreshold perceptual processes, such as temporal processing, can show a stronger link with cognition. The reviewed evidence underlines the importance of fully accounting for perceptual deficits when investigating cognitive decline in old age.

## Introduction

**Figure 1:**
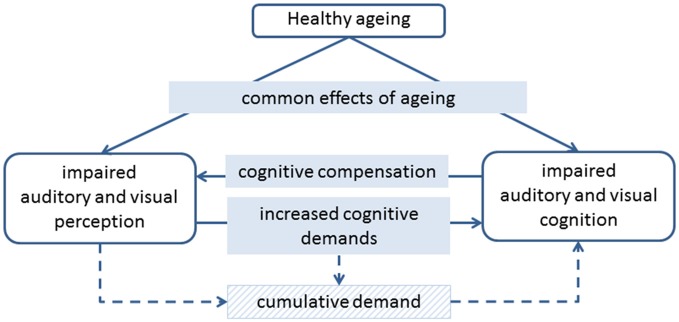
**Diagram of the potential links between healthy ageing, auditory and visual perception, and auditory and visual cognition**.

It is well known that ageing is associated with declines in both perception and cognition. As we age, there is increased need for perceptual aids such as glasses and hearing aids, and we start to find cognitive tasks such as paying attention and remembering more difficult. It is possible that perception and cognition decline in tandem due to common effects of ageing, but there is also evidence to suggest that declines in perception and cognition impact on each other, both in the short and long term (Figure 1). Here we review evidence that impoverished perceptual input can increase the cognitive difficulty of tasks, while changes to cognitive strategies and processes can compensate, to some extent, for impaired perception. We also review evidence that both visual and auditory perceptual impairments are associated with a faster rate of cognitive decline, and consider whether perceptual aids, such as hearing aids and glasses, can provide protection against cognitive decline.

## Age-Related Declines in Hearing, Vision and Cognition

The ability to hear faint sounds in a quiet environment deteriorates with age. Peripheral hearing sensitivity, as measured by the audiogram, is impaired in around a third of 61–70 year olds and almost two thirds of over-70 year olds (Davis, 1989; Cruickshanks et al., 1998; Wilson et al., 1999). Ageing can also affect suprathreshold auditory processing. Sensitivity to temporal fine structure is impaired in older adults, even in those with normal audiometric thresholds (Grose and Mamo, 2010; Füllgrabe, 2013; Füllgrabe et al., 2015), as is sensitivity to changes in the temporal envelope (Füllgrabe et al., 2015).

Vision also declines in old age. As with hearing, many of these changes are peripheral, and other changes affect central processing. Hardening of the lens leads to presbyopia, which makes it more difficult to focus on near objects. As well as age-related eye diseases such as cataracts, glaucoma and macular degeneration, healthy ageing is linked to a thickening and yellowing of the lens (Said and Weale, 1959; Ruddock, 1965). Ageing is also associated with changes in color perception (Page and Crognale, 2005), temporal resolution (Wright and Drasdo, 1985; Kim and Mayer, 1994; Culham and Kline, 2002), visual acuity (Spear, 1993), motion perception (Snowden and Kavanagh, 2006; Hutchinson et al., 2012), and a loss of fine detail (high spatial frequency) pattern vision (Elliott, 1987; Pardhan, 2004).

Deficits in one sensory modality can sometimes be offset through other senses, e.g., using visual speechreading to support impaired hearing (Dickinson and Taylor, 2011), or increased multisensory integration (Freiherr et al., 2013). This may be limited in older age when both hearing and vision decline, particularly as declines in vision and audition appear to be linked, with higher than expected rates of dual-sensory decline (Dawes et al., 2014).

Old age also brings decline in a number of cognitive abilities, including working memory, memory, attention, and executive control (Schaie, 1996; Hedden and Gabrieli, 2004). Cross-sectional and longitudinal studies consistently find that ageing is related to poor performance on tasks involving cognitive control and switching, manipulation of information, visuospatial processing, processing speed and more (for an extensive list of studies, see Hofer et al., 2003). There are a number of proposed explanations for this decline, including generalized slowing (e.g., Salthouse, 1996) and dedifferentiation (e.g., Wilson et al., 2012). In this brief review, we focus on the role of sensory function.

A number of cross-sectional, population-based studies have demonstrated a link between (auditory and visual) sensory impairment and poor cognition in old age (Lindenberger and Baltes, 1994; Baltes and Lindenberger, 1997; Lin et al., 2004, 2011; Tay et al., 2006; Moore et al., 2014), that does not simply reflect the visibility or audibility of the task materials (Lindenberger et al., 2001). The association between perception and cognition is also present in younger adults, but is stronger in older adults (Baltes and Lindenberger, 1997). While the evidence for a link between perception and cognition is compelling, it is not universally found. Two studies have found a link between vision and cognition but not hearing and cognition (Anstey et al., 2001; Gussekloo et al., 2005), one study failed to find a link between hearing and cognition (Gennis et al., 1991), and one found no link between visual or auditory function and cognition in a narrow-age cohort sample of 75 year olds (Hofer et al., 2003).

The majority of studies that have attempted to link sensory and cognitive decline have used only visual (or auditory) acuity as a proxy for more general perceptual decline (e.g., Anstey et al., 2001; Hofer et al., 2003). If a common factor underlies age-related changes in performance, then we would expect changes to also affect more central, suprathreshold perceptual skills. However, few studies have used more higher order, cortical measures of perception. Humes et al. (2013) used an index of temporal sensory processing and found a clear link to performance on temporal cognitive tasks. Glass (2007) found that visual contrast sensitivity was a better predictor of performance in cognitive tasks with unfamiliar, multiple or complex stimuli than simple stimuli. These results suggest that going beyond simple sensory acuity may reveal a more direct link to cognitive task performance.

Various hypotheses have been proposed to account for the link between perception and cognition (Table 1; see Wayne and Johnsrude, 2015 for a recent review of these hypotheses in the auditory modality). Here we consider both vision and audition as well as possible effects of compensatory cognition.

**Table 1:** **Proposed hypotheses for the link between perceptual and cognitive decline in old age**.

Hypothesis	Description
Common cause	A third, general factor underlies the decline in both perception and cognition (Lindenberger and Baltes, 1994; Baltes and Lindenberger, 1997).
Cognitive load on perception	Poor cognition results in poor performance on perceptual tests (Lindenberger and Baltes, 1994).
Information degradation	Impoverished perceptual input impacts on performance on cognitive tasks (Schneider and Pichora-Fuller, 2000).
Sensory deprivation	Over time, impoverished perceptual input leads to cognitive decline (Lindenberger and Baltes, 1994).

*Note that these hypotheses are not mutually exclusive*.

## Effects of Ageing influence Both Perception and Cognition (“Common Cause”)

Sensory systems are subject to the same molecular and cellular processes as the rest of the body and as such, any general age-related change will affect both sensory and cognitive mechanisms (e.g., Pathai et al., 2013). Cardiovascular risk factors, for example, are associated with both hearing loss (Shargorodsky et al., 2010) and cognitive decline (Knopman et al., 2001).

The link between sensory and cognitive impairment is typically found after adjusting for age, suggesting that it is not simply an effect of general ageing. In a detailed study, Humes et al. (2013) found that the link between age and cognitive function was entirely mediated by sensory function, based on a composite measure of auditory, visual and tactile perception. It therefore seems that while common effects of ageing do affect both perception and cognition, it is likely that perception and cognition directly impact on each other (Figure 1).

## Poor Cognition Affects Performance on Perceptual Tasks (“Cognitive Load on Perception”)

Perception and cognition are highly interrelated, such that even measures that might be considered to be entirely sensory, such as the audiogram, have been shown to be influenced by cognition (Zwislocki et al., 1958). More complex perceptual tasks are likely to be more strongly influenced by the participant’s cognitive abilities. While it is important that researchers consider the impact of cognition on their perceptual measures, it seems unlikely that poor cognition drives purely perceptual decline. Schneider and Pichora-Fuller (2000) report empirical evidence of this, based on a study that showed age-related impairment for some perceptual tasks, but not others, despite cognitive demands being held constant across conditions (Pichora-Fuller and Schneider, 1991). In contrast, there is strong evidence that cognition has a compensatory role in adapting to impaired perception (see below and Figure 1). A more likely causal link between perception and cognition suggests that impoverished perceptual input impacts on cognitive function, both directly and in the longer term.

## Impoverished Perceptual input Directly Impacts Cognitive Resources (“information Degradation”)

When the perceptual signal is poor, either through degraded stimuli or impaired perception, additional cognitive resources are required to decipher the signal. For example, cognitive load in listening tasks, as measured by the pupil response, is higher for those with hearing loss (Zekveld et al., 2011). This leaves fewer cognitive resources available for performing the cognitive task. A number of studies have demonstrated that recall of target words is impaired if the words are degraded, either through the addition of noise or hearing impairment, even when ensuring that the words can still be identified (e.g., Rabbitt, 1968, 1991; Pichora-Fuller et al., 1995; McCoy et al., 2005; Tun et al., 2009; Ng et al., 2013). This is generally considered to be evidence that the additional effort required to decode degraded stimuli takes up cognitive resources that would otherwise be involved in encoding and rehearsal (Rabbitt, 1968, 1991). Similar effects are found in young and older adults, and it is not yet clear whether ageing confers an additional deficit, over and above that of perceptual loss. Several studies have confounded age and hearing impairment by comparing young, normally-hearing adults with older, hearing-impaired adults (e.g., Pichora-Fuller et al., 1995; Mishra et al., 2014). Verhaegen et al. (2014) found that young and older adults with matched hearing impairment showed similar recall performance on a verbal short-term memory task, which was worse than that found in young adults with normal hearing. On the other hand, the link between working memory capacity and speech-in-noise perception has been shown to be weaker for young adults than normally-hearing older adults (Füllgrabe and Rosen, 2016) who differed in their sensitivity to temporal-fine-structure information (Füllgrabe, 2013).

Recently there has been a concerted effort to describe and quantify the loss of cognitive resources when the perceptual input is degraded (Rudner and Lunner, 2014). This includes development of a “cognitive spare capacity” (CSC) test (Mishra et al., 2013), which is a measure of auditory working memory incorporating both storage and executive processing. Using this task under a variety of listening conditions, Mishra et al. (2014) found that older, hearing-impaired adults had similar CSC to young, normally-hearing adults when listening conditions were optimal. This again suggests that task difficulty in old age can relate to sensory, rather than cognitive, impairment.

It is worth noting that all of the above evidence comes from auditory studies; the impact of impoverished visual input on visual cognition has been less thoroughly explored. Visual sensory quality has been suggested as an explanation for age-related changes in performance of the Stroop task (Ben-David and Schneider, 2009, 2010), providing a sensory explanation for what has previously been considered a change in (cognitive) inhibition.

There is also evidence that hearing impairment affects performance on visual tasks. Rönnberg et al. (2014) found that older adults with hearing loss had worse performance on visuospatial short- and long-term memory (LTM) tasks. Similarly, an earlier study found a link between hearing loss and LTM function when the LTM task required motor encoding (Rönnberg et al., 2011). These findings point to more general effects of sensory loss than simply increasing the difficulty of cognitive tasks. Rönnberg et al. (2011) hypothesize that LTM representations are used less by people with hearing loss due to a mismatch between the input signal and the stored signal, and that this disuse leads to a decline in LTM over time.

## Sensory Impairment Leads to Cognitive Decline (“Sensory Deprivation”)

A handful of longitudinal studies have shown that impaired perception is associated with cognitive decline, providing support for a causal link between perceptual and cognitive decline over time (Lin et al., 2004, 2013; Ghisletta and Lindenberger, 2005). For example, women with impaired (corrected) vision at baseline had a faster rate of cognitive decline over a 4 year period than those without visual impairment (Lin et al., 2004). In addition, Lin et al. (2013) found that poor hearing at baseline was associated with a higher rate of cognitive impairment and a faster rate of cognitive decline over a 6 year period. In contrast, Anstey et al. (2001) found a link between visual, but not auditory, decline and cognition over a 2 year period, and Valentijn et al. (2005) found that although there was a link between perceptual and cognitive decline, there was no convincing evidence that baseline auditory and visual acuity predicted cognitive decline over the following 6 years. The current evidence is therefore mixed, but it is important to note that only the effects of peripheral sensory changes, such as acuity, have been considered. There appears to be a stronger link with cognition when measures of perception go beyond simple sensory acuity (e.g., Humes et al., 2013).

Several plausible mechanisms have been proposed to explain how impaired perception could lead to worsening cognition over time (e.g., Lin et al., 2013). One possibility is that poor perception leads to social isolation (Strawbridge et al., 2000), which in turn leads to cognitive decline. However, Dawes et al. (2015) found that while hearing-aid use was associated with better cognition, this was not mediated by social isolation.

A further possibility is that performance on cognitive tasks is reduced by the ongoing effort from sensory deprivation (cf. Rönnberg et al., 2011). In this case, the ongoing effort puts a strain on cognition, which eventually leads to performance breakdown. This hypothesis predicts that short-term improvements in stimulus quality (or perceptual abilities) will have limited effectiveness, but that longer-term improvements in perception will help maintain cognition.

There is not yet convincing evidence to suggest that hearing aids or glasses offer protection against cognitive decline. Hearing-aid use is generally associated with better cognition (Lin, 2011; Rönnberg et al., 2014; Dawes et al., 2015), but it is possible that the people who seek treatment differ from those who do not, both cognitively and functionally. Two studies have shown no cognitive benefit from cataract surgery (Hall et al., 2005; Valentijn et al., 2005) or hearing aids (Valentijn et al., 2005). A randomized controlled trial of hearing-aid use showed a positive change in communication, social and emotional function, and depression, but no longer-term improvement in cognition (Mulrow et al., 1990, 1992). There is therefore no compelling evidence that correcting for peripheral sensory deficits improves cognition.

## Cognition Can Compensate for The Effects of Impoverished Perceptual input

While a lack-of-use explanation may account well for effects of poor perception on memory function, it is less clear how this might extend to other cognitive skills such as executive control. It has been widely established that older adults use compensatory cognitive strategies to deal with, among other things, impoverished perceptual input. It is not yet clear if perception-related cognitive decline occurs as a function of the increased cognitive demands, or despite them.

Older adults can engage compensatory cognitive processes in order to perform at a similar level to younger adults. This is reflected in different patterns of cortical activity (Reuter-Lorenz and Lustig, 2005) and is associated with better performance compared to older adults who show less compensatory activation (Cabeza et al., 2002). For example, when listening to speech, older adults showed reduced activation in auditory cortex, but increased activation in prefrontal and precuneus regions associated with working memory and attention (Wong et al., 2009). The increased activation in cognitive regions was associated with better behavioral performance, although older adults were still at a disadvantage relative to younger adults when the signal-to-noise ratio was unfavorable.

Few studies have investigated the direct effect of sensory degradation on compensatory mechanisms. There is some evidence that increasing the difficulty of encoding the perceptual task can lead to increased compensatory activity. For example, Schulte et al. (2011) manipulated perceptual and cognitive demands in a match-to-sample Stroop task. Older adults showed a different pattern of fronto-parietal and visuomotor activation, indicating recruitment of additional regions to cope with increased cognitive and perceptual difficulty. This suggests that increasing visual complexity increases the requirement for compensatory activity.

Recruitment of additional cortical regions is usually linked to maintained performance on cognitive tasks (e.g., Schulte et al., 2011). As task difficulty increases, however, performance can collapse. In a letter memory task, older adults had similar performance to younger adults, but showed widespread additional cortical activations (in comparison to younger adults). As task difficulty increased, younger adults also showed these additional activations. At the highest task difficulty the performance of older adults declined, as did their brain activations (Schneider-Garces et al., 2010). This is consistent with a peak or ceiling for performance, which falls with age. The longer term consequences of repeatedly reaching this ceiling are not yet clear.

## Conclusions and Future Directions

There is clear evidence for a link between perception and cognition in old age, in terms of both their impact on task performance and their age-related decline. While there are clearly common and general factors acting on both sensory and cognitive decline, there also appears to be a more direct link between impaired perception and cognitive decline. Degraded input leads to a higher load on cognition, reducing resources available for cognitive processing. It has been proposed that, over time, this sensory deprivation leads to cognitive decline. At the same time, compensatory processes have been shown that allow people to ameliorate the effects of age-related perceptual decline.

There is therefore something of a paradox, that while staying cognitively active can protect against cognitive decline in old age (Hultsch et al., 1999), needing to be more cognitively active to overcome poor perceptual input is associated with cognitive decline. It should be noted that the majority of evidence for the sensory deprivation hypothesis comes from the auditory domain, whereas evidence for compensatory processes comes predominantly from the visual domain. Future research in both domains could draw on findings from the other modality.

A further avenue for future work is to consider what is evaluated when assessing sensory abilities. Where perception has been linked to cognition, typically only sensory acuity has been assessed. Where measures have gone beyond simple sensory acuity, a clearer link to cognitive change can often be seen.

Improving the quality of the perceptual input should reduce cognitive impairment in the short term and reduce the involvement of compensatory mechanisms. In the longer term, there is little suggestion that cataract surgery and hearing aids can improve cognition. This may simply reflect limits on the number of longitudinal studies and their follow-up periods, but it may also indicate that interventions should be aimed at more central perceptual processes. An alternative approach would be to investigate whether improving cognition through (for example) brain training could have a beneficial effect on auditory or visual perception. Differentiating between stimulation-based interventions and compensatory cognitive training (Kim and Kim, 2014) could provide further insights into the mechanism that links perceptual and cognitive decline in older age.

## Author Contributions

KLR and HAA reviewed the literature and wrote the article.

## Conflict of Interest Statement

The authors declare that the research was conducted in the absence of any commercial or financial relationships that could be construed as a potential conflict of interest.

## References

[B1] Anstey K. J. Luszcz M. A. Sanchez L. (2001). Two-year decline in vision but not hearing is associated with memory decline in very old adults in a population-based sample. Gerontology 47, 289–293. 10.1159/000052814 11490149

[B2] Baltes P. B. Lindenberger U. (1997). Emergence of a powerful connection between sensory and cognitive functions across the adult life span: a new window to the study of cognitive aging? Psychol. Aging 12, 12–21. 10.1037/0882-7974.12.1.12 9100264

[B3] Ben-David B. M. Schneider B. A. (2009). A sensory origin for color-word Stroop effects in aging: a meta-analysis. Neuropsychol. Dev. Cogn. B Aging Neuropsychol. Cogn. 16, 505–534. 10.1080/13825580902855862 19479479

[B4] Ben-David B. M. Schneider B. A. (2010). A sensory origin for color-word Stroop effects in aging: simulating age-related changes in color-vision mimics age-related changes in Stroop. Neuropsychol. Dev. Cogn. B Aging Neuropsychol. Cogn. 17, 730–746. 10.1080/13825585.2010.510553 21058053

[B5] Cabeza R. Anderson N. D. Locantore J. K. McIntosh A. R. (2002). Aging gracefully: compensatory brain activity in high-performing older adults. Neuroimage 17, 1394–1402. 10.1006/nimg.2002.1280 12414279

[B6] Cruickshanks K. J. Wiley T. L. Tweed T. S. Klein B. E. Klein R. Mares-Perlman J. A.. (1998). Prevalence of hearing loss in older adults in Beaver Dam, Wisconsin. The epidemiology of hearing loss study. Am. J. Epidemiol. 148, 879–886. 10.1093/oxfordjournals.aje.a009713 9801018

[B7] Culham J. C. Kline D. W. (2002). The age deficit on photopic counterphase flicker: contrast, spatial frequency and luminance effects. Can. J. Exp. Psychol. 56, 177–186. 10.1037/h0087395 12271748

[B8] Davis A. C. (1989). The prevalence of hearing impairment and reported hearing disability among adults in Great Britain. Int. J. Epidemiol. 18, 911–917. 10.1093/ije/18.4.911 2621028

[B9] Dawes P. Dickinson C. Emsley R. Bishop P. N. Cruickshanks K. J. Edmondson-Jones M.. (2014). Vision impairment and dual sensory problems in middle age. Ophthalamic Physiol. Opt. 34, 479–488. 10.1111/opo.12138 24888710 PMC4273649

[B10] Dawes P. Emsley R. Cruickshanks K. J. Moore D. R. Fortnum H. Edmondson-Jones M.. (2015). Hearing loss and cognition: the role of hearing aids, social isolation and depression. PLoS One 10:e0119616. 10.1371/journal.pone.0119616 25760329 PMC4356542

[B11] Dickinson C. M. Taylor J. (2011). The effect of simulated visual impairment on speech-reading ability. Ophthalmic Physiol. Opt. 31, 249–257. 10.1111/j.1475-1313.2010.00810.x 21410739

[B12] Elliott D. B. (1987). Contrast sensitivity decline with aging: a neural or optical phenomenon. Ophthalamic Physiol. Opt. 7, 415–419. 10.1016/0275-5408(87)90065-2 3454919

[B13] Freiherr J. Lundström J. N. Habel U. Reetz K. (2013). Multisensory integration mechanisms during aging. Front. Hum. Neurosci. 7:863. 10.3389/fnhum.2013.00863 24379773 PMC3861780

[B14] Füllgrabe C. (2013). Age-dependent changes in temporal-fine-structure processnig in the absence of peripheral hearing loss. Am. J. Audiol. 22, 313–315. 10.1044/1059-0889(2013/12-0070) 23975124

[B16] Füllgrabe C. Moore B. C. J. Stone M. A. (2015). Age-group differences in speech identification despite matched audiometrically normal hearing: contributions from auditory temporal processing and cognition. Front. Aging Neurosci. 6:347. 10.3389/fnagi.2014.00347 25628563 PMC4292733

[B15] Füllgrabe C. Rosen S. (2016). “Investigating the role of working memory in speech-in-noise identification for listeners with normal hearing,” in Physiology, Psychoacoustics and Cognition in Normal and Impaired Hearing, eds van Dijk P. Baskent D. Gaudrain E. de Kleine E. Wagner A. Lanting C. (Heidelberg: Springer), 29–36. 10.1007/978-3-319-25474-6_4 PMC5714061 27080643

[B17] Gennis V. Garry P. J. Haaland K. Y. Yeo R. A. Goodwin J. S. (1991). Hearing and cognition in the elderly. New findings and a review of the literature. Arch. Intern. Med. 151, 2259–2264. 10.1001/archinte.151.11.2259 1953231

[B18] Ghisletta P. Lindenberger U. (2005). Exploring structural dynamics within and between sensory and intellectual functioning in old and very old age: longitudinal evidence from the berlin aging study. Intelligence 33, 555–587. 10.1016/j.intell.2005.07.002

[B19] Glass J. M. (2007). Visual function and cognitive aging: differential role of contrast sensitivity in verbal versus spatial tasks. Psychol. Aging 22, 233–238. 10.1037/0882-7974.22.2.233 17563179

[B20] Grose J. H. Mamo S. K. (2010). Processing of temporal fine structure as a function of age. Ear Hear. 31, 755–760. 10.1097/AUD.0b013e3181e627e7 20592614 PMC2966515

[B21] Gussekloo J. de Craen A. J. M. Oduber C. van Boxtel M. P. J. Westendorp R. G. J. (2005). Sensory impairment and cognitive functioning in oldest-old subjects: the Leiden 85+ Study. Am. J. Geriatr. Psychiatry 13, 781–786. 10.1176/appi.ajgp.13.9.781 16166407

[B22] Hall T. A. McGwin G. Jr. Owsley C. (2005). Effect of cataract surgery on cognitive function in older adults. J. Am. Geriatr. Soc. 53, 2140–2144. 10.1111/j.1532-5415.2005.00499.x 16398899

[B23] Hedden T. Gabrieli J. D. E. (2004). Insights into the ageing mind: a view from cognitive neuroscience. Nat. Rev. Neurosci. 5, 87–97. 10.1038/nrn1323 14735112

[B24] Hofer S. M. Berg S. Era P. (2003). Evaluating the interdependence of aging-related changes in visual and auditory acuity, balance and cognitive functioning. Psychol. Aging 18, 285–305. 10.1037/0882-7974.18.2.285 12825777

[B25] Hultsch D. F. Hertzog C. Small B. J. Dixon R. A. (1999). Use it or lose it: engaged lifestyle as a buffer of cognitive decline in aging? Psychol. Aging 14, 245–263. 10.1037/0882-7974.14.2.245 10403712

[B26] Humes L. E. Busey T. A. Craig J. Kewley-Port D. (2013). Are age-related changes in cognitive function driven by age-related changes in sensory processing? Atten. Percept. Psychophys. 75, 508–524. 10.3758/s13414-012-0406-9 23254452 PMC3617348

[B27] Hutchinson C. V. Arena A. Allen H. A. Ledgeway T. (2012). Psychophysical correlates of global motion processing in the aging visual system: a critical review. Neurosci. Biobehav. Rev. 36, 1266–1272. 10.1016/j.neubiorev.2012.02.009 22343109

[B29] Kim E. Y. Kim K. W. (2014). A theoretical framework for cognitive and non-cognitive interventions for older adults: stimulation versus compensation. Aging Mental Health 18, 304–315. 10.1080/13607863.2013.868404 24354740

[B28] Kim C. B. Y. Mayer M. J. (1994). Foveal flicker sensitivity in healthy aging eyes. J. Opt. Soc. Am. A Opt. Image Sci. Vis. 11, 1958–1969. 10.1364/josaa.11.001958 8071737

[B30] Knopman D. Boland L. L. Mosley T. Howard G. Liao D. Szklo M.. (2001). Cardiovascular risk factors and cognitive decline in middle-aged adults. Neurology 56, 42–48. 10.1212/wnl.56.1.42 11148234

[B31] Lin F. R. (2011). Hearing loss and cognition among older adults in the United States. J. Gerontol. A Biol. Sci. Med. Sci. 66A, 1131–1136. 10.1093/gerona/glr115 21768501 PMC3172566

[B33] Lin F. R. Ferrucci L. Metter E. J. An Y. Zonderman A. B. Resnick S. M. (2011). Hearing loss and cognition in the baltimore longitudinal study of ageing. Neuropsychology 25, 763–770. 10.1037/a0024238 21728425 PMC3193888

[B32] Lin F. R. Gutierrez P. R. Stone K. L. Yaffe K. Ensrud K. E. Fink H. A.. (2004). Vision impairment and combined vision and hearing impairment predict cognitive and functional decline in older women. J. Am. Geriatr. Soc. 52, 1996–2002. 10.1111/j.1532-5415.2004.52554.x 15571533

[B34] Lin F. R. Yaffe K. Xia J. Xue Q. L. Harris T. B. Purchase-Helzner E.. (2013). Hearing loss and cognitive decline in older adults. JAMA Intern. Med. 173, 293–299. 10.1001/jamainternmed.2013.1868 23337978 PMC3869227

[B35] Lindenberger U. Baltes P. B. (1994). Sensory functioning and intelligence in old age: a strong connection. Psychol. Aging 9, 339–355. 10.1037/0882-7974.9.3.339 7999320

[B36] Lindenberger U. Scherer H. Baltes P. B. (2001). The strong connection between sensory and cognitive performance in old age: not due to sensory reductions operating during cognitive assessment. Psychol. Aging 16, 196–205. 10.1037/0882-7974.16.2.196 11405308

[B37] McCoy S. L. Tun P. A. Cox L. C. Colangelo M. Stewart R. A. Wingfield A. (2005). Hearing loss and perceptual effort: downstream effects on older adults’ memory for speech. Q. J. Exp. Psychol. A 58A, 22–33. 10.1080/02724980443000151 15881289

[B38] Mishra S. Lunner T. Stenfelt S. Rönnberg J. Rudner M. (2013). Visual information can hinder working memory processing of speech. J. Speech Lang. Hear. Res. 56, 1120–1132. 10.1044/1092-4388(2012/12-0033) 23785180

[B39] Mishra S. Stenfelt S. Lunner T. Rönnberg J. Rudner M. (2014). Cognitive spare capacity in older adults with hearing loss. Front. Aging Neurosci. 6:96. 10.3389/fnagi.2014.00096 24904409 PMC4033040

[B40] Moore D. R. Edmondson-Jones M. Dawes P. Fortnum H. McCormack A. Pierzycki R. H.. (2014). Relation between speech-in-noise threshold, hearing loss and cognition from 40–69 years of age. PLoS One 9:e107720. 10.1371/journal.pone.0107720 25229622 PMC4168235

[B41] Mulrow C. D. Aguilar C. Endicott J. E. Tuley M. R. Velez R. Charlip W. S.. (1990). Quality-of-life changes and hearing impairment: a randomized trial. Ann. Intern. Med. 113, 188–194. 10.7326/0003-4819-113-3-188 2197909

[B42] Mulrow C. D. Tuley M. R. Aguilar C. (1992). Sustained benefits of hearing-aids. J. Speech Hear. Res. 35, 1402–1405. 10.1044/jshr.3506.1402 1494282

[B43] Ng E. H. N. Rudner M. Lunner T. Pedersen M. S. Rönnberg J. (2013). Effects of noise and working memory capacity on memory processing of speech for hearing-aid users. Int. J. Audiol. 52, 433–441. 10.3109/14992027.2013.776181 23550584

[B44] Page J. W. Crognale M. A. (2005). Differential aging of chromatic and achromatic visual pathways: behavior and electrophysiology. Vision Res. 45, 1481–1489. 10.1016/j.visres.2004.09.041 15743617

[B45] Pardhan S. (2004). Contrast sensitivity loss with aging: sampling efficiency and equivalent noise at different spatial frequencies. J. Opt. Soc. Am. A Opt. Image Sci. Vis. 21, 169–175. 10.1364/josaa.21.000169 14763759

[B46] Pathai S. Shiels P. G. Lawn S. D. Cook C. Gilbert C. (2013). The eye as a model of ageing in translational research - molecular, epigenetic and clinical aspects. Ageing Res. Rev. 12, 490–508. 10.1016/j.arr.2012.11.002 23274270

[B47] Pichora-Fuller M. K. Schneider B. A. (1991). masking-level differences in the elderly: a comparison of antiphasic and time-delay dichotic conditions. J. Speech Hear. Res. 34, 1410–1422. 10.1044/jshr.3406.1410 1787722

[B48] Pichora-Fuller M. K. Schneider B. A. Daneman M. (1995). How young and old adults listen to and remember speech in noise. J. Acoust. Soc. Am. 97, 593–608. 10.1121/1.412282 7860836

[B50] Rabbitt P. M. A. (1968). Channel-capacity intelligibility and immediate memory. Q. J. Exp. Psychol. A 20, 241–248. 10.1080/14640746808400158 5683763

[B49] Rabbitt P. (1991). Mild hearing-loss can cause apparent memory failures which increase with age and reduce with IQ. Acta Otolaryngol. Suppl. 476, 167–176. 10.3109/00016489109127274 2087959

[B51] Reuter-Lorenz P. A. Lustig C. (2005). Brain aging: reorganizing discoveries about the aging mind. Curr. Opin. Neurobiol. 15, 245–251. 10.1016/j.conb.2005.03.016 15831410

[B52] Rönnberg J. Danielsson H. Rudner M. Arlinger S. Sternäng O. Wahlin Å.. (2011). Hearing loss is negatively related to episodic and semantic long-term memory but not to short-term memory. J. Speech Lang. Hear. Res. 54, 705–726. 10.1044/1092-4388(2010/09-0088) 20884779

[B53] Rönnberg J. Hygge S. Keidser G. Rudner M. (2014). The effect of functional hearing loss and age on long- and short-term visuospatial memory: evidence from the UK biobank resource. Front. Aging Neurosci. 6:326. 10.3389/fnagi.2014.00326 25538617 PMC4260513

[B54] Ruddock K. H. (1965). The effect of age upon colour vision II. Changes with age in light transmission of the ocular media. Vision Res. 5, 47–58. 10.1016/0042-6989(65)90074-x 5862761

[B55] Rudner M. Lunner T. (2014). Cognitive spare capacity and speech communication: a narrative overview. Biomed Res. Int. 2014:869726. 10.1155/2014/869726 24971355 PMC4058272

[B56] Said F. S. Weale R. A. (1959). The variation with age of the spectral transmissivity of the living human crystalline lens. Gerontologia 3, 213–231. 10.1159/000210900 14440799

[B57] Salthouse T. A. (1996). The processing-speed theory of adult age differences in cognition. Psychol. Rev. 103, 403–428. 10.1037/0033-295x.103.3.403 8759042

[B58] Schaie K. W. (1996). Intellectual Development in Adulthood: The Seattle Longitudinal Study. Cambridge: Cambridge University Press.

[B59] Schneider B. A. Pichora-Fuller M. K. (2000). “Implications of perceptual deterioration for cognitive aging research,” in The Handbook of Aging and Cognition, 2nd Edn, eds Craik F. I. M. Salthouse T. A. (London: Lawrence Erlbaum Associates), 155–219.

[B60] Schneider-Garces N. J. Gordon B. A. Brumback-Peltz C. R. Shin E. Lee Y. Sutton B. P.. (2010). Span, CRUNCH and beyond: working memory capacity and the aging brain. J. Cogn. Neurosci. 22, 655–669. 10.1162/jocn.2009.21230 19320550 PMC3666347

[B61] Schulte T. Müller-Oehring E. M. Chanraud S. Rosenbloom M. J. Pfefferbaum A. Sullivan E. V. (2011). Age-related reorganization of functional networks for successful conflict resolution: a combined functional and structural MRI study. Neurobiol. Aging 32, 2075–2090. 10.1016/j.neurobiolaging.2009.12.002 20022675 PMC2888896

[B62] Shargorodsky J. Curhan S. G. Eavey R. Curhan G. C. (2010). A prospective study of cardiovascular risk factors and incident hearing loss in men. Laryngoscope 120, 1887–1891. 10.1002/lary.21039 20715090 PMC3968532

[B63] Snowden R. J. Kavanagh E. (2006). Motion perception in the ageing visual system: minimum motion, motion coherence and speed discrimination thresholds. Perception 35, 9–24. 10.1068/p5399 16491704

[B64] Spear P. D. (1993). Neural bases of visual deficits during aging. Vision Res. 33, 2589–2609. 10.1016/0042-6989(93)90218-l 8296455

[B65] Strawbridge W. J. Wallhagen M. I. Shema S. J. Kaplan G. A. (2000). Negative consequences of hearing impairment in old age: a longitudinal analysis. Gerontologist 40, 320–326. 10.1093/geront/40.3.320 10853526

[B66] Tay T. Wang J. J. Kifley A. Lindley R. Newall P. Mitchell P. (2006). Sensory and cognitive association in older persons: findings from an australian population. Gerontology 52, 386–394. 10.1159/000095129 16921251

[B67] Tun P. A. McCoy S. Wingfield A. (2009). Aging, hearing acuity and the attentional costs of effortful listening. Psychol. Aging 24, 761–766. 10.1037/a0014802 19739934 PMC2773464

[B68] Valentijn S. A. M. van Boxtel M. P. van Hooren S. A. Bosma H. Beckers H. J. Ponds R. W.. (2005). Change in sensory functioning predicts change in cognitive functioning: results from a 6-year follow-up in the maastricht aging study. J. Am. Geriatr. Soc. 53, 374–380. 10.1111/j.1532-5415.2005.53152.x 15743277

[B69] Verhaegen C. Collette F. Majerus S. (2014). The impact of aging and hearing status on verbal short-term memory. Neuropsychol. Dev. Cogn. B Aging Neuropsychol. Cogn. 21, 464–482. 10.1080/13825585.2013.832725 24007209

[B70] Wayne R. V. Johnsrude I. S. (2015). A review of causal mechanisms unerlying the link between age-related hearing loss and cognitive decline. Ageing Res. Rev. 23, 154–166. 10.1016/j.arr.2015.06.002 26123097

[B72] Wilson R. S. Segawa E. Hizel L. P. Boyle P. A. Bennett D. A. (2012). Terminal dedifferentiation of cognitive abilities. Neurology 78, 1116–1122. 10.1212/WNL.0b013e31824f7ff2 22491858 PMC3320052

[B71] Wilson D. H. Walsh P. G. Sanchez L. Davis A. C. Taylor A. W. Tucker G.. (1999). The epidemiology of hearing impairment in an australian adult population. Int. J. Epidemiol. 28, 247–252. 10.1093/ije/28.2.247 10342686

[B73] Wong P. C. M. Jin J. X. Gunasekera G. M. Abel R. Lee E. R. Dhar S. (2009). Aging and cortical mechanisms of speech perception in noise. Neuropsychologia 47, 693–703. 10.1016/j.neuropsychologia.2008.11.032 19124032 PMC2649004

[B74] Wright C. E. Drasdo N. (1985). The influence of age on the spatial and temporal contrast sensitivity fucntion. Doc. Ophthalmol. 59, 385–395. 10.1007/bf00159172 4028926

[B75] Zekveld A. A. Kramer S. E. Festen J. M. (2011). Cognitive load during speech perception in noise: the influence of age, hearing loss and cognitionon the pupil response. Ear Hear. 32, 498–510. 10.1097/AUD.0b013e31820512bb 21233711

[B76] Zwislocki J. Maire F. Feldman A. S. Rubin H. (1958). On the effect of practice and motivation on the threshold of audibility. J. Acoust. Soc. Am. 30, 254–262. 10.1121/1.1909559

